# Adsorption of CO on α‐Al_2_O_3_(0001): A Combined Experimental and Computational Study

**DOI:** 10.1002/cphc.202401134

**Published:** 2025-04-17

**Authors:** Siddhi Gojare, Shuang Chen, Jiachen Chen, Zairan Yu, Juana Vázquez Quesada, Philipp N. Pleßow, Karin Fink, Yuemin Wang

**Affiliations:** ^1^ Institute of Nanotechnology (INT) Karlsruher Institut für Technologie (KIT) Kaiserstraße 12 76131 Karlsruhe Germany; ^2^ Institute of Functional Interfaces (IFG) Karlsruhe Institute of Technology (KIT) Kaiserstraße 12 76131 Karlsruhe Germany; ^3^ Institute of Catalysis Research and Technology (IKFT) Karlsruhe Institute of Technology (KIT) Kaiserstraße 12 76131 Karlsruhe Germany

**Keywords:** atomic structures, density functional theory, IR spectroscopy, surface chemistry, α‐Al_2_O_3_

## Abstract

α‐Al_2_O_3_ is a widely utilized material with diverse technological applications, particularly as a catalyst support in heterogeneous catalysis. Here, a systematic investigation of the interaction between CO and the α‐Al_2_O_3_(0001) single‐crystal surface is presented by combining polarization‐resolved infrared reflection absorption spectroscopy with theoretical calculations. The latter includes a comprehensive analysis of multiple coverage scenarios using periodic density functional theory calculations, as well as various embedded quantum cluster models to evaluate the performance of hybrid functionals and wavefunction methods such as MP2. The combined results reveal that the Al‐terminated α‐Al_2_O_3_(0001) surface exhibits high reactivity and is stabilized by partial hydroxylation even under ultrahigh vacuum conditions. This is evidenced by two characteristic CO bands (2172–2195 cm^−1^ for CO‐Al^3+^ and 2163 cm^−1^ for OH^…^CO) with distinct binding energies, which are consistent with theoretical predictions.

## Graphical abstract

The comprehensive results obtained by combining polarization‐resolved IRRAS with periodic DFT calculations and various embedded quantum cluster models provide solid evidence that the α‐Al_2_O_3_(0001) surface is stabilized by partial hydroxylation, characterized by two distinct CO vibrations at 2172–2195 cm^−1^ (CO–Al^3+^) and 2163 cm^−1^ (OH^…^CO ) with different binding energies.

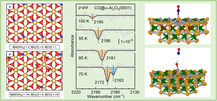

## 1 Introduction

α‐Al_2_O_3_ (corundum) is an important ceramic known for its excelent mechanical properties. It is used in a wide range of fields, including optical windows, thin‐film microelectronics, and as an advanced substrate for ultrathin metal films and semiconductors.^[^ ^1^, ^2^, ^3^, ^4^, ^5^, ^6^, ^7^ ^]^ In heterogeneous catalysis, it is widely utilized primarily as a catalyst support, playing a crucial role in many important industrial catalytic reactions, such as the conversion of CH_4_ to syngas^[^ ^8^ ^]^ and Fischer–Tropsch synthesis.^[^ ^9^, ^10^ ^]^ The surface chemistry of α‐Al_2_O_3_ has been extensively investigated through numerous experimental and theoretical approaches with the aim of elucidating its surface structure and chemical reactivity.^[^ ^11^, ^12^, ^13^, ^14^, ^15^, ^16^, ^17^, ^18^, ^19^, ^20^, ^21^, ^22^ ^]^ Given the great complexity of Al_2_O_3_ nanomaterials, a thorough understanding remains a challenging task. Consequently, surface science studies based on well‐controlled model systems are required to provide reliable and accurate reference data. Infrared spectroscopy (IR) has the advantage that it can be applied to metal oxide systems, in the form of both macroscopic monocrystals and powder particles.^[^ ^23^, ^24^ ^]^ However, studies using infrared reflection absorption spectroscopy (IRRAS) on oxide single‐crystal surfaces face significant inherent experimental challenges due to the dielectric properties of oxidic substrates.

The α‐Al_2_O_3_(0001) surface is known to be the energetically most stable surface of α‐Al_2_O_3_. The bare, adsorbate‐free α‐Al_2_O_3_(0001) has been proposed to be single Al‐terminated. Here, we present the polarization‐resolved IRRAS data on the α‐Al_2_O_3_(0001) single‐crystal surface using CO as a probe molecule. The CO surface‐ligand IR (CO‐SLIR) approach has been demonstrated to be extremely sensitive to the surface structure of catalysts and the chemical environments of adsorption sites.^[^ ^25^, ^26^, ^27^, ^28^, ^29^ ^]^ The IRRAS characterization was complemented by grazing‐emission X‐ray photoelectron spectroscopy (XPS). The assignment of CO vibrations was aided by systematic theoretical investigations, including periodic density functional theory (DFT) calculations and embedded cluster computations. We provide direct spectroscopic evidence for the partial hydroxylation of the α‐Al_2_O_3_(0001) surface even under ultrahigh vacuum (UHV) conditions. Two distinct CO vibrational bands are unambiguously identified in the p‐polarized IRRAS data (2163 cm^−1^ for OH^…^CO and 2172–2195 cm^−1^ for CO‐Al^3+^) with characteristic binding energies. These experimental findings are supported by a comprehensive theoretical analysis.

## 2 Results

### 2.1 XPS and IRRAS Data

**Figure 1:**
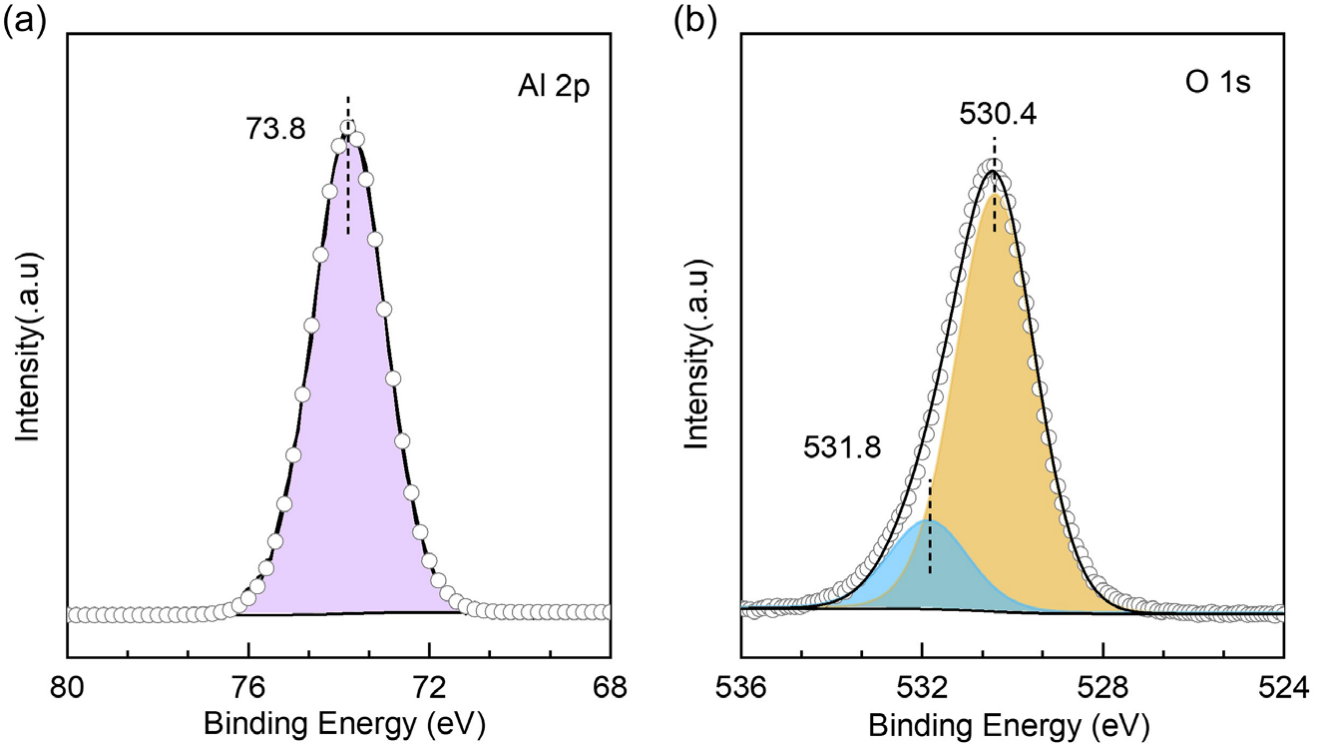
Grazing‐emission XPS characterization of the freshly prepared α‐Al_2_O_3_(0001) surface under UHV conditions: a) Al 2*p* core level spectrum and b) O 1*s* core level spectrum.

The clean α‐Al_2_O_3_(0001) surface was initially assessed using grazing‐emission XPS, a technique known for its high surface sensitivity.^[^ ^30^ ^]^ This method enables gaining valuable information regarding the elemental composition and chemical states at surfaces. The deconvoluted XPS data of Al 2*p* (73.8 eV for Al^3+^) and O 1*s* core levels are shown in **Figure** **1**. In addition to the prominent O 1*s* peak at 530.4 eV, attributed to regular lattice oxygen O^2−^, a secondary component at 531.8 eV is resolved, characteristic of hydroxyl species. Based on a quantitative analysis, the surface concentration of OH groups is estimated to be ≈18%. These results indicate that the pristine α‐Al_2_O_3_(0001) surface, prepared according to the well‐established preparation procedures in UHV outlined in the experimental section, is not free of adsorbates but rather partially hydroxylated.

**Figure 2:**
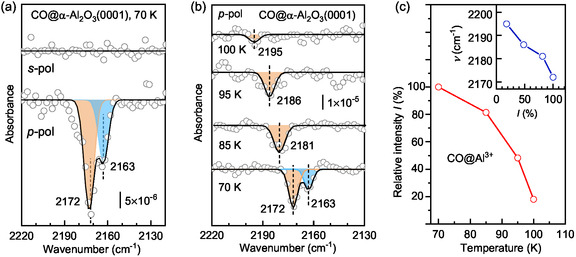
a) Polarization‐resolved IRRAS data recorded after 5 L CO adsorption on α‐Al_2_O_3_(0001) at 70 K at a grazing incidence angle of 80°. b) Temperature‐dependent IRRAS data obtained after saturation adsorption of CO at 70 K on the α‐Al_2_O_3_(0001) surface and subsequently annealing to indicated temperatures. c) Intensity evolution of the CO‐Al^3+^ band as a function of sample temperature; the insert shows that the attenuation of the CO‐Al^3+^ band is accompanied by a gradual blue shift in frequency.

Partial hydroxylation of the α‐Al_2_O_3_(0001) surface is further confirmed by the corresponding IRRAS data. **Figure** **2**a displays the deconvoluted, polarization‐resolved IRRAS results obtained after exposing the pristine α‐Al_2_O_3_(0001) surface to 5 L of CO at 70 K (saturation adsorption). In the p‐polarized spectrum, two distinct CO vibrations are clearly identified at 2163 and 2172 cm^−1^. The dominant band at 2172 cm^−1^ is attributed to CO adsorbed on surface Al^3+^ cations as the majority species, whereas the weaker signal at 2163 cm^−1^ is typical for CO weakly bound to acidic hydroxyl groups via (OH^…^CO) hydrogen bonding.^[^ ^31^, ^32^, ^33^, ^34^ ^]^ The assignment of these CO bands is validated by theoretical results obtained using periodic DFT calculations and various embedded quantum cluster (EQC) models, as described in Section 2.2, 3.

It is important to note that for oxide single crystals serving as dielectric substrates, the IR bands of adsorbates can exhibit significant variations in both sign and intensity.^[^ ^23^, ^30^ ^]^ This variability arises from the interaction of the vibrational transition dipole moment with different components of the incident IR light: p‐polarized light parallel (*E*_p,t_) and perpendicular (*E*_p,n_) to the substrate surface as well as the s‐polarized component (*E*_s_). In the IRRAS data recorded with s‐polarized light (Figure 2a), the absence of vibrational signals indicates that both CO species adopt a nearly upright adsorption geometry.

The temperature‐dependent IRRAS data provide solid evidence of two distinct CO species with different binding energies. As shown in Figure 2b, the IR band at 2163 cm^−1^ disappears completely when the sample temperature is slightly increased to 85 K, indicating a weakly bound CO species. This observation aligns with its assignment to OH^…^CO species stabilized via hydrogen bonding. In contrast, the 2172 cm^−1^ band gradually decreases in intensity with further heating and vanishes around 100 K (Figure 2b,c), suggesting relatively higher thermal stability, consistent with CO species chemisorbed to surface Al^3+^ cations. Using the Redhead equation^[^ ^35^ ^]^ and assuming a pre‐exponential factor of 10^13^ s^−1^, the binding energies for low‐coverage CO species were estimated to be ≈25 ± 2 kJ mol^−1^ (0.26 ± 0.02 eV) for OH^…^CO and 30 ± 2 kJ mol^−1^ (0.31 ± 0.02 eV) for CO–Al^3+^, respectively.

Interestingly, the attenuation of the CO–Al^3+^‐related IR signal, that is, a decrease in coverage, is accompanied by a significant blue shift in frequency, from 2172 cm^−1^ at 70 K to 2195 cm^−1^ at 100 K (Figure 2c). This coverage‐induced frequency shift has been observed for CO adsorption on other oxide surfaces (e.g., ZnO^[^ ^36^ ^]^ and Y‐stabilized ZrO_2_^[^ ^29^ ^]^) and can be explained in terms of the repulsive lateral adsorbate–adsorbate interactions, involving both dynamic and substrate‐mediated static interactions.^[^ ^36^ ^]^

Overall, the polarization‐resolved IRRAS and grazing‐emission XPS results reveal that the stoichiometric α‐Al_2_O_3_(0001) surface exhibits high reactivity and is stabilized by partial hydroxylation with surface OH groups present even under UHV conditions, consistent with previous theoretical predications.^[^ ^37^ ^]^ These experimental findings are further supported by systematic theoretical investigations, as described in the following.

### 2.2 Theoretical Results

#### 2.2.1 Periodic DFT Calculations

**Figure 3:**
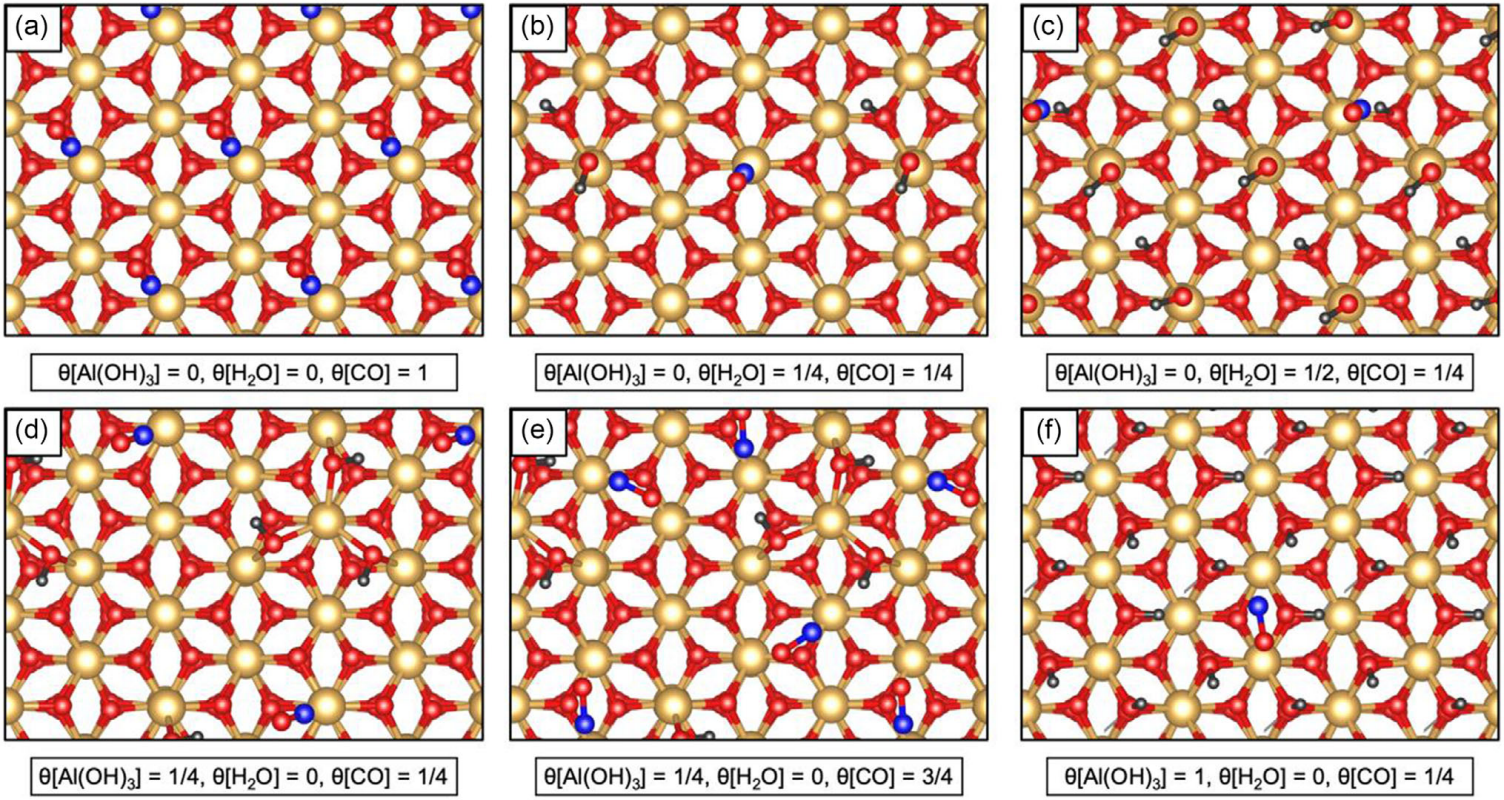
Configurations of CO adsorption on different α‐Al_2_O_3_(0001) surfaces. Each structure a–f) is described by the coverage rate of Al(OH)_3_, H_2_O, and CO. The yellow sphere represents the Al atom, red represents O, black represents H, and blue represents C. All configurations are displayed in top views.

Different adsorption configurations were investigated for various CO coverage rates on *α*‐Al_2_O_3_(0001) surfaces, including dry (stoichiometric), water‐dissociated, and partially and fully hydroxylated surfaces. The typical configurations are shown in **Figure** **3**. Here, each adsorption configuration is described by the degree of surface hydroxylation (*θ*[Al(OH)_3_]), the water coverage (*θ*[H_2_O]), and the CO coverage (*θ*[CO]).

**Figure 4:**
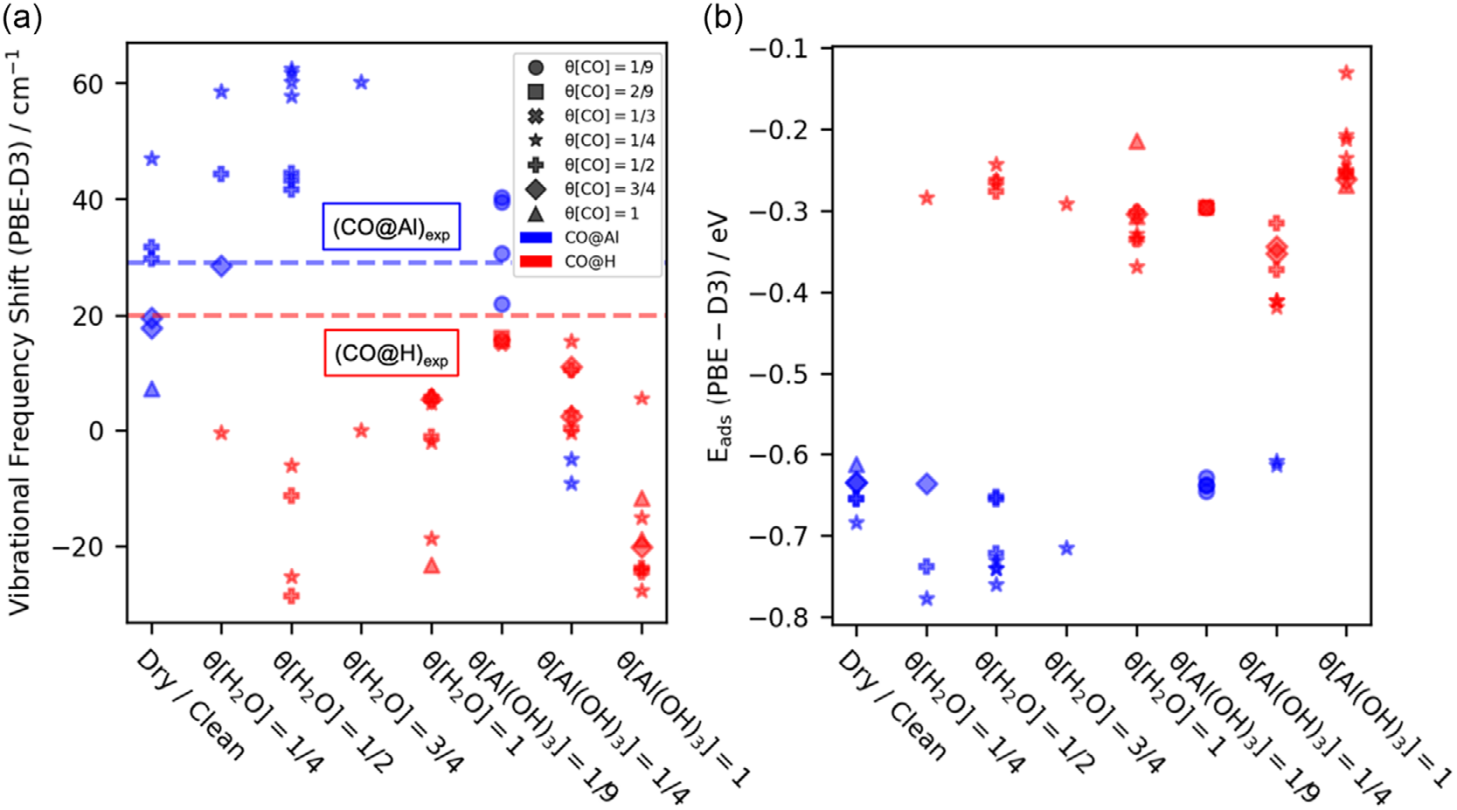
a) Calculated vibrational frequency shift and b) adsorption energy of various CO adsorption rates on different α‐Al_2_O_3_(0001) surfaces. The different markers represent different CO coverage rates, and the colors represent the adsorption sites. The blue and red dashed lines are the vibrational frequency shift of CO adsorbed on Al and H, respectively, obtained from the experiments at 70 K.

**Figure** **4**a shows the vibrational frequency shift obtained by subtracting the computed vibrational frequency of CO adsorbed on the surface to the calculated vibrational frequency of CO in vacuum (2124.3 cm^−1^) using the PBE(D3) level of theory. The most important factor affecting the vibrational frequency is the adsorption site. CO adsorbed on Al typically exhibits a higher frequency while adsorption on H leads to a lower frequency, which agrees with the experimental data (see Figure 2a). Multiple vibrational frequencies are observed when more than one CO per unit cell is studied, where each CO bound in a different environment contributes one unique frequency. In case of degenerate or almost degenerate vibrations, only the vibration of the most stable CO is shown.

**Figure 5:**
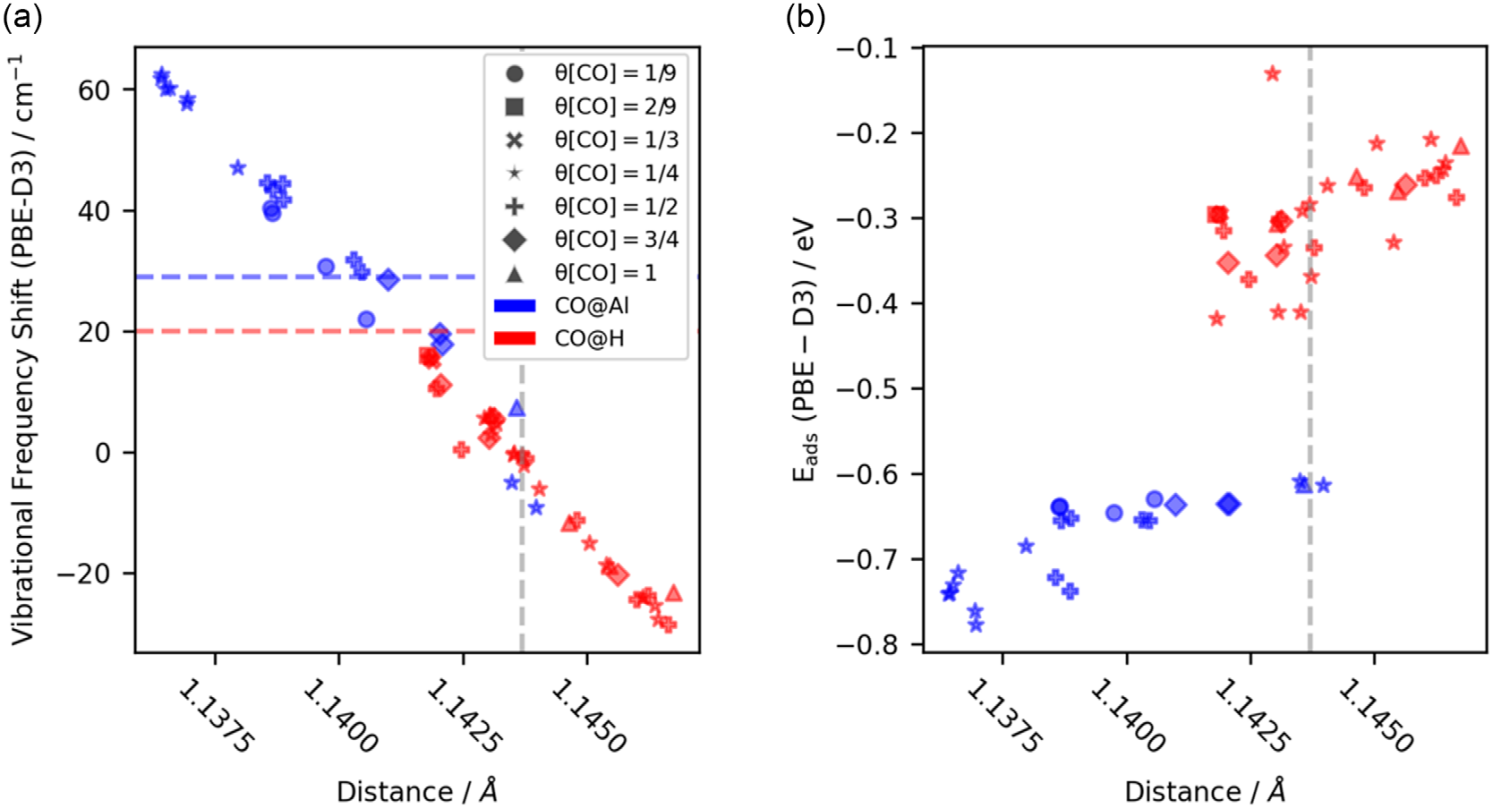
a) CO bond length versus vibrational frequency shift (see text). The vertical gray dashed line represents the computational value of the bond length of free CO. The blue and red dashed lines are the vibrational frequency shift of CO adsorbed on Al and H, respectively, obtained from the experiments at 70 K. b) CO bond length versus adsorption energy.

Adsorption energies are shown in Figure 4b. The binding of CO on Al is at least 0.2 eV stronger than for adsorption on H. For low CO coverages, such as 1/4 monolayer (ML), the presence of H_2_O (*θ*[H_2_O] = 1/4, *θ*[H_2_O] = 1/2, *θ*[H_2_O] = 3/4 ML, see Figure 4b) will enhance the strength of CO adsorption on Al sites relative to the dry surface. However, this enhancement is gradually weakened with the increase of H_2_O coverage being nonexistent by *θ*[H_2_O] = 1 ML for all the CO coverages. The presence of Al(OH)_3_ will undermine the CO adsorption on the Al site [*θ*[Al(OH_3_)] = 1/9 > *θ*[Al(OH_3_)] = 1/4 > *θ*[Al(OH_3_)] = 1 ML]. A linear relationship between bond length and vibrational frequency shift is presented in **Figure** **5**a. It is observed that the shorter the CO bond distance, the larger the blue shift of the vibrational frequency for the adsorbed CO with respect to the CO in the gas phase. Figure 5b shows the CO adsorption energy as a function of the CO bond length. While CO molecules bound to an Al show stronger binding and shorter bonds than CO bound to H, there is a correlation within these two groups.

**Figure 6:**
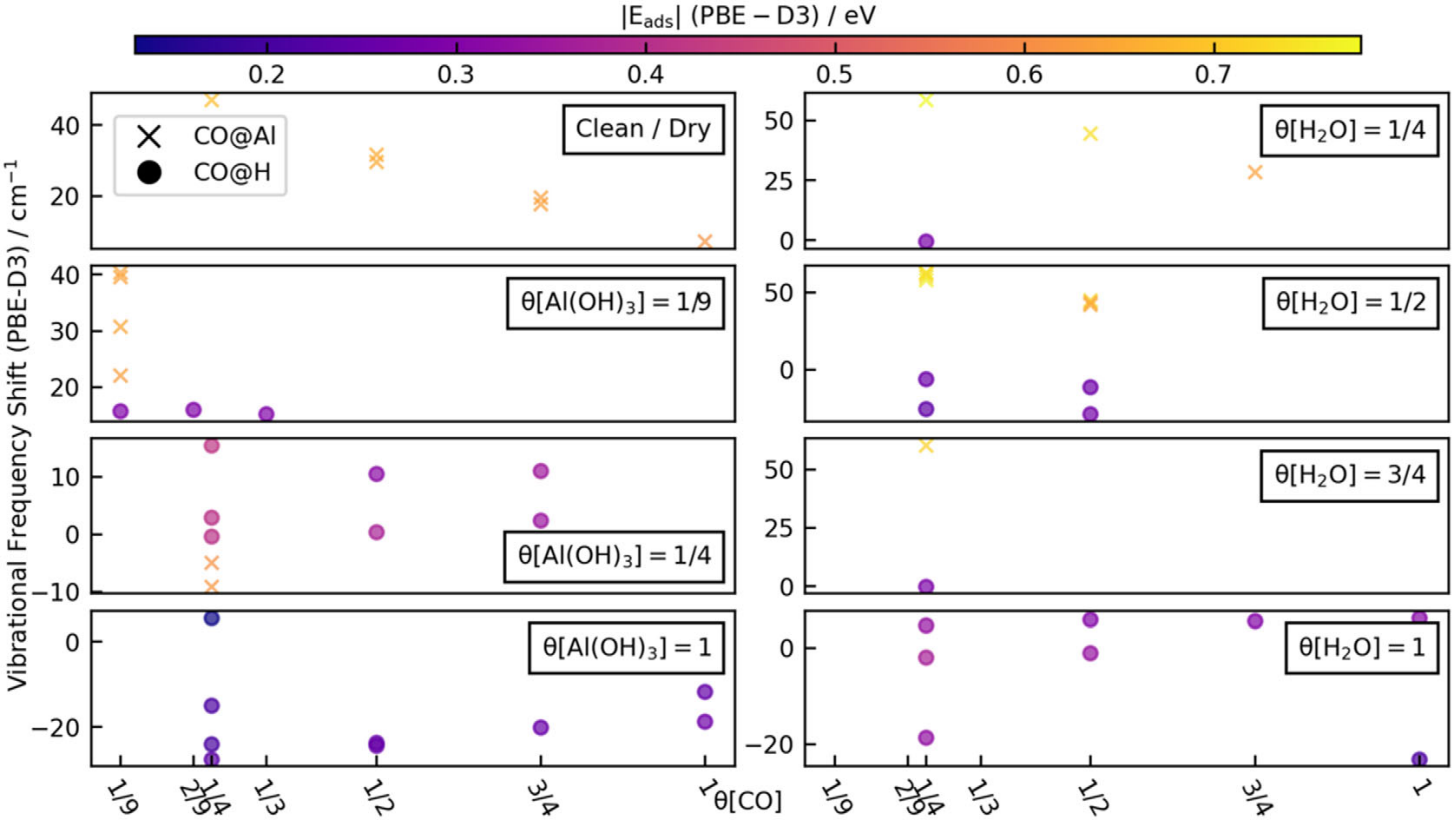
Variation of CO vibrational frequency shift as a function of CO coverage (ML) on different surfaces. The marker color indicates the adsorption energy and corresponds to the color bar.

The effects of CO coverage on the CO vibrational frequency shift are shown in **Figure** **6**. On the dry surface, Al is the only available site, and all the vibrational frequencies are blue‐shifted. As CO coverage increases, the vibrational frequency decreases almost linearly. On *θ*[Al(OH)_3_] = 1/9 ML, the vibrational frequencies of CO on both Al and H sites are blue‐shifted. For CO adsorption on Al, the adsorption site (position relative to Al(OH)_3_) and angle determine the vibration frequency. For the H site, CO is adsorbed on the hydroxyl group of Al(OH)_3_, and the number of CO does not affect the vibrational frequency. For surfaces with a higher degree of hydroxylation, *θ*[Al(OH)_3_] = 1/4 ML, the CO vibrational frequency shift of CO adsorption on H decreases with increasing CO coverage. There is only one available Al site on this surface, which is the surface Al farthest from *θ*[Al(OH)_3_]. We found two stable adsorption configurations with different adsorption angles but similar adsorption energies (≈0.61 eV) for this adsorption site. For the fully hydroxylated surface, *θ*[Al(OH)_3_] = 1 ML, there is no exposed Al site; therefore, all CO can only be adsorbed on H. In an extremely weakly adsorbed configuration, the vibrational frequency will be blue‐shifted. The vibrational frequencies will be red‐shifted for other relatively more stable adsorption configurations (*E*_ads_ = 0.20–0.25 eV). For the stable adsorption configuration, the vibrational frequency increases with the CO coverage.

For surfaces with water molecules dissociated (Figure 6, right panel), as the water coverage increases, the available H sites increase, while the Al sites decrease. For *θ*[H_2_O] = 1/4 ML, adsorption on H hardly changes the vibrational frequency of CO. However, when CO is adsorbed on Al, the vibrational frequency shift increases further compared to when it is adsorbed on Al on a dry surface. A similar phenomenon also occurs on *θ*[H_2_O] = 1/2 ML. On this surface, CO adsorption on H produces a red shift but is insensitive to CO coverage. Since each CO is not identical when more CO is adsorbed on the *θ*[H_2_O] = 3/4 ML, we only list the results of 1 CO adsorption here. For *θ*[H_2_O] = 1 ML, the vibrational frequencies at different adsorption positions are quite different, with CO adsorbed on lower positions of OH having lower vibrational frequencies. At the same time, the vibrational frequency is almost unaffected by the CO coverage.

**Figure 7:**
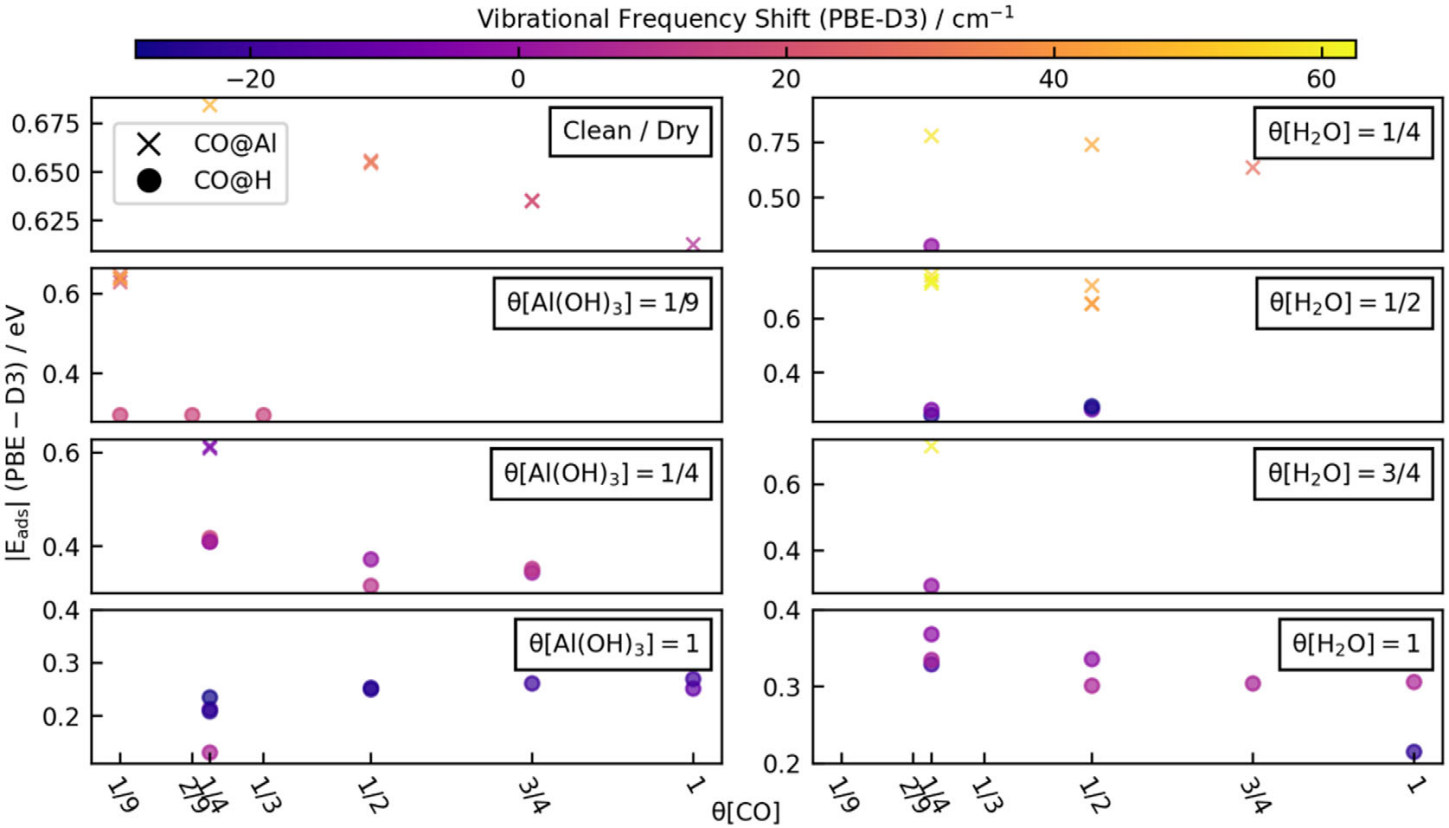
Variation of CO BE as a function of CO coverage (ML) on different surfaces. The marker color indicates the vibrational frequency shift (see text) and corresponds to the color bar.

The effect of CO coverage on the BE is displayed in **Figure** **7**. On the dry surface, the adsorption energy becomes weaker as the CO coverage increases. For surfaces with different degrees of hydroxylation, the adsorption energy of CO is almost unaffected by the CO coverage. For water‐dissociated surfaces, the adsorption energy of CO decreases with increasing CO coverage. Meanwhile, the presence of water enhances the adsorption of CO on Al sites.

#### 2.2.2 Embedded Cluster DFT Calculations

The periodic DFT computations focused mainly on the effect of CO coverage with respect to the different hydroxylation levels of the α‐Al_2_O_3_(0001) surface using PBE‐D3 level of theory. The local interaction of one CO molecule with the α‐Al_2_O_3_(0001) surface describing the case of low CO coverage was analyzed using an embedded cluster model. The embedded quantum cluster (EQC) approach enabled also the study of the performance of hybrid functionals, such as B3LYP and PBE0, as well as the wavefunction‐based method MP2. The α‐Al_2_O_3_(0001) surface with the surface terminations, *θ*[H_2_O] = 0 ML (water‐free, dry surface), *θ*[Al(OH)_3_] = 1 ML level of hydroxylation, and the dissociated H_2_O (*θ*[H_2_O] = 1 ML) along with a single adsorbed CO molecule were considered to investigate the aforementioned aspects.

**Figure 8:**
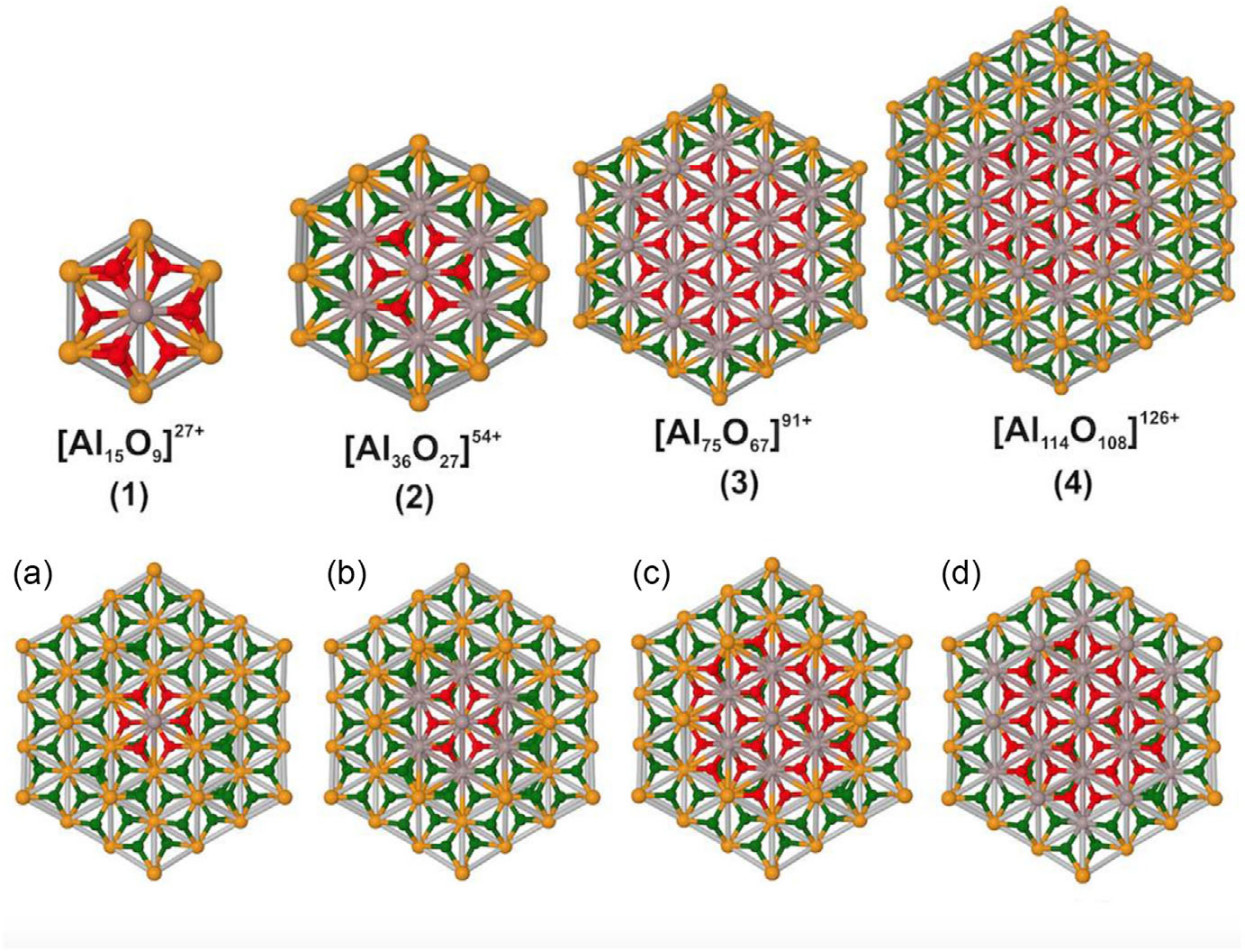
Different cluster sizes for the water‐free (clean/dry, *θ*[H_2_O] = 0 ML) α‐Al_2_O_3_(0001) surface (1–4) considered in this work and (a–d) schematics of the quantum cluster (3) showing sequential relaxed Al and O shells surrounding the adsorption site of the CO molecule. Color scheme of atoms: yellow—fixed Al, gray—unfixed Al, green—fixed oxygen, red—unfixed oxygen.

For the choice of the model cluster, two key factors were considered to ensure the necessary accuracy: the cluster size and the parts of the cluster to be structurally relaxed. The CO binding energy (BE)—without basis‐set superposition error (BSSE) correction—was used to monitor these two aspects. By this means, various cluster sizes of the water‐free surface (see **Figure** **8**(1–4)) were explored by consecutively adding surrounding Al and O shells. The analysis revealed that the CO BE converged gradually until sizes (3) and (4) with BE of the order of −0.95 and −0.97 eV (see Table S1, Supporting Information for more details). Therefore, all further calculations were carried out on cluster (3).

Regarding the influence of the structural relaxation, sequential shell relaxations of the cluster (3) (schematically shown for the water‐free surface in Figure 8a–d) were investigated. The considered relaxed shells are represented by gray color for Al and red color for O in this figure. In the first relaxed shell geometry, only the central Al and the first oxygen shell (6 atoms) were relaxed while keeping the rest fixed. The relaxation shells were systematically increased by consecutively adding Al and O shells to the already relaxed cluster part. The atoms at the boundary region of the cluster were always kept fixed (see Figure 8).

**Figure 9:**
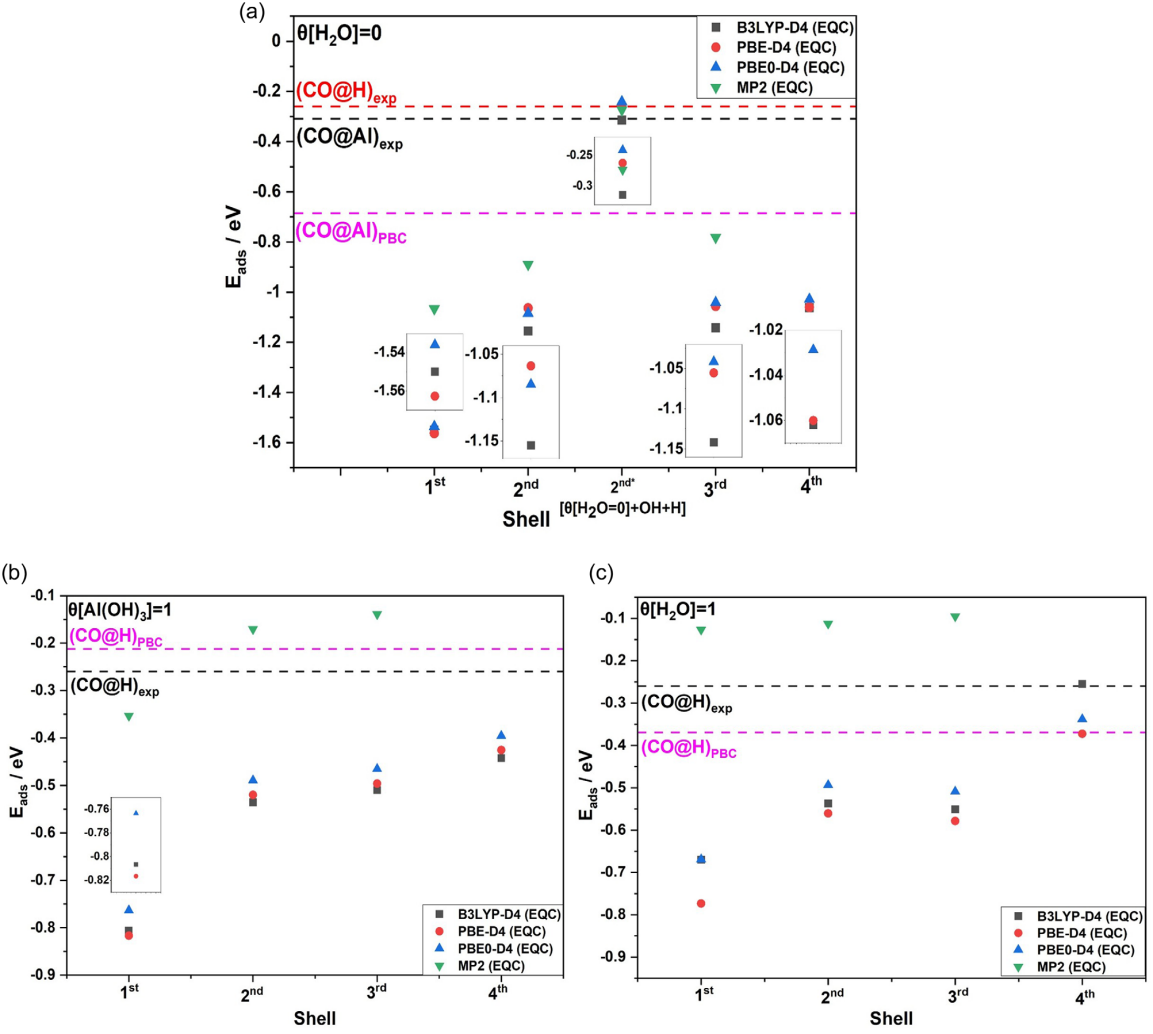
CO binding energy as a function of the number of relaxed shells of cluster size (3) obtained for a) the water‐free (*θ*[H_2_O] = 0 ML) surface including the results for the model *θ*[[H_2_O = 0] + OH + H] with CO adsorbed on the hydroxyl group denoted 2nd* *θ*[[H_2_O = 0] + OH + H] (see text for further description), as well as for b) full hydroxylated (*θ*[Al(OH)_3_] = 1 ML) and c) H_2_O dissociated (*θ*[H_2_O] = 1 ML) surfaces. In all the cases, only a single CO molecule was adsorbed. The horizontal dashed lines represent the binding energies obtained by periodic DFT (PBE‐D3, low CO coverage) and in the experiment. The def2‐TZVPP basis set was used in all the computations.

The variation of the CO BE—without correction of the BSSE—as a function of the number of relaxed shells is displayed in **Figure** **9**. If only the first relaxation shell is considered, the BE is strongly overestimated. Relaxations of the 2nd shell lead to a strong reduction, while there are only small changes by inclusion of the 3rd shell for the *θ*[H_2_O] = 0, *θ*[Al(OH)_3_] = 1, and *θ*[H_2_O] = 1 ML surfaces. Thus, considering the data convergence and the computational cost, it was chosen to continue working with relaxation of cluster (3) until the 3rd shell. So far, the BSSE correction was not considered for the convergence tests but is taken into account for the final binding energies (see **Table** **1** and discussion section).

**Table 1:** Summary of the results for CO binding energy [eV] and CO vibrational frequency shift Δ(ν(CO)) [cm^−1^] on two different sites: (i) an Al site at *θ*[H_2_O] = 0 ML with a single CO molecule in the embedded quantum cluster (EQC) approach and *θ*[CO] = 1/4 ML in the periodic boundary conditions (PBC) calculations and (ii) an OH site for low OH termination, described by the *θ*[[H_2_O = 0] + OH + H] EQC and PBC calculations at *θ*[Al(OH)_3_] = 1/9 ML and *θ*[CO] = 1/9 ML.

	CO properties		Method					
		Exp.	PBC		EQCc)			
			PBE(D3)	PBE0(D4)	PBE(D4)	B3LYP(D4)	PBE0(D4)	MP2
*θ*[H_2_O] = 0 (CO on Al)	BE/eV	−0.31	−0.68a)	−0.66b)	−0.55	−0.75	−0.60	−0.39
	Δν(co)/cm^−1^	52^(100 K)^	47	–	40	72	65	81
*θ*[[H_2_O = 0] + OH + H] (CO on OH)	BE/eV	−0.26	−0.3d)	–	−0.22	−0.27	−0.20	−0.21
	Δν(co)/cm^−1^	19^(70 K)^	15d)	–	12	17	17	18

a) The value was obtained for the water‐free surface with a CO coverage of *θ*[CO] = 1/4 ML (see Figure 6). A single point calculation at the PBE(D4) level on the PBE(D3) structures yielded a binding energy of −0.69 eV.

b) The binding energy at the PBE0(D4) level was calculated using relaxed structures obtained at the PBE(D3) level.

c) The binding energies for EQC calculations were corrected for the BSSE using the full CP scheme (see text). For the water‐free *θ*[H_2_O] = 0 ML surface and in the case of the DFT computations, the full CP correction was carried out using a grid size of m4 and a convergence threshold of 1.0 × 10^−8^ for the density and SCF equations.

d) For CO adsorption on top of an OH group, the PBC calculations correspond to the low‐coverage case (*θ*[Al(OH)_3_] = 1/9 ML; *θ*[CO] = 1/9 ML), as shown in Table S7, Supporting Information.

**Figure 10:**
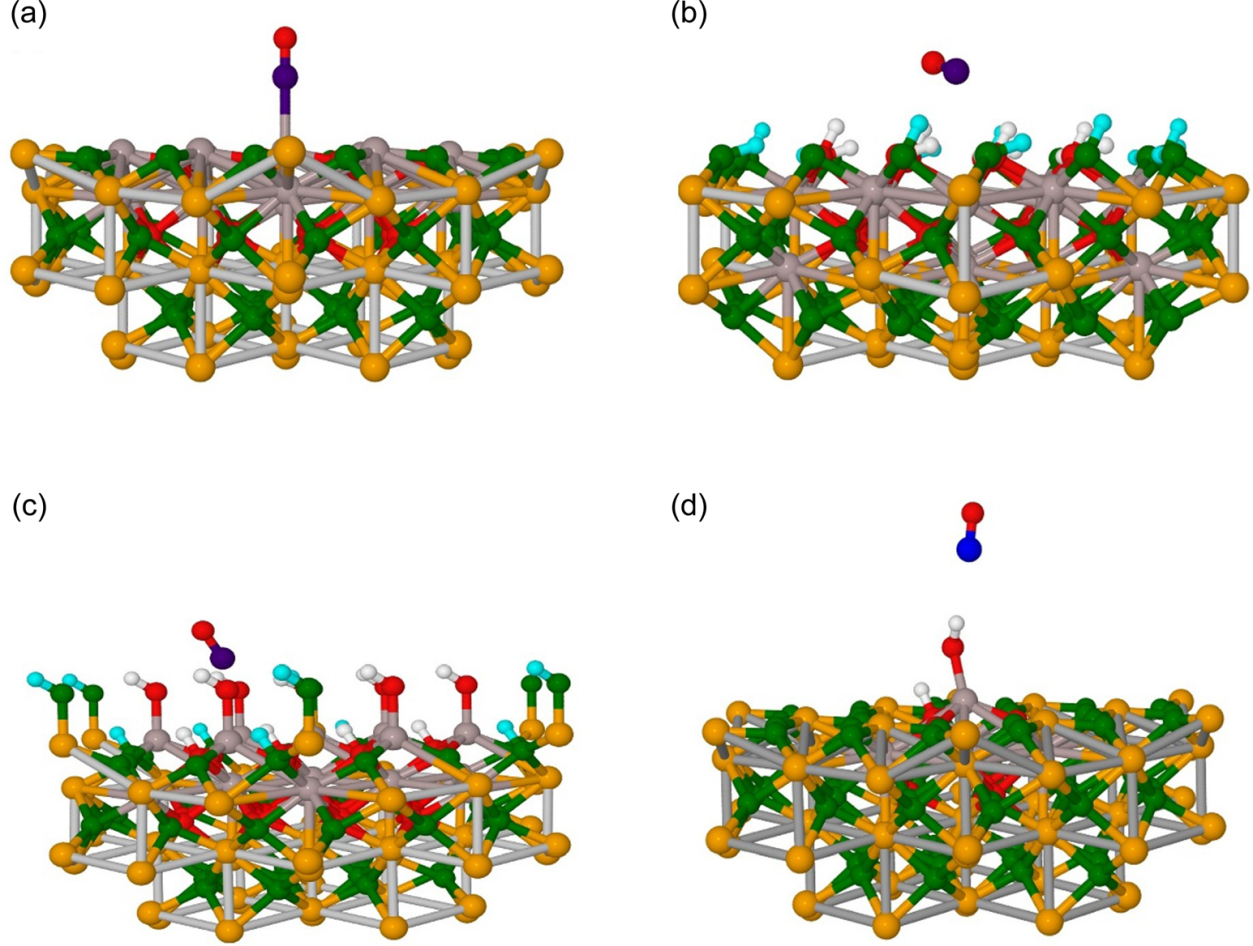
Schematic of the side view of CO binding configurations on different α‐Al_2_O_3_(0001) surfaces: a) water‐free (clean/dry, *θ*[H_2_O] = 0 ML), b) fully hydroxylated (*θ*[Al(OH)_3_] = 1 ML), c) H_2_O dissociated (*θ*[H_2_O] = 1 ML), and d) low‐coverage model (*θ*[[H_2_O = 0] + OH + H], see text). Color scheme of atoms: yellow—fixed Al, gray—unfixed Al, green—fixed oxygen, red—unfixed oxygen, blue—carbon, white—unfixed hydrogen, cyan—fixed hydrogen.

**Figure 11:**
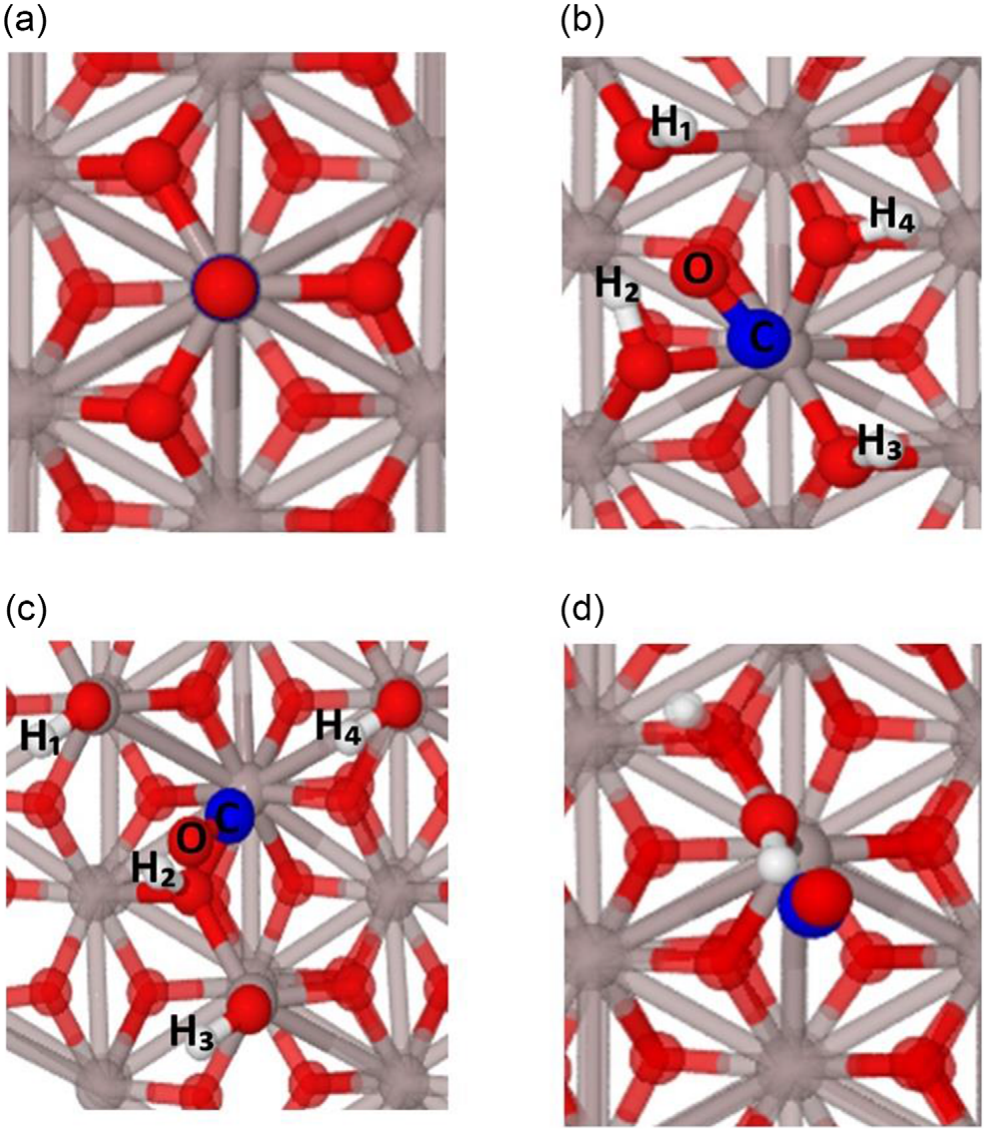
Top view of the clusters presented in Figure 10 showing the interaction between CO molecules and the nearby surface hydroxyl groups. a) Water‐free (clean/dry, *θ*[H_2_O] = 0) surface, b) fully hydroxylated (*θ*[Al(OH)_3_ = 1 ML] surface, c) H_2_O dissociated (*θ*[H_2_O] = 1 ML) surface, and d) low‐coverage model (*θ*[[H_2_O = 0] + OH + H], see text). The H_1_–H_4_ in (b) and (c) denote the neighboring hydroxyl groups with which the hydrogen bond distance was calculated. Color scheme of atoms: gray for Al, red for oxygen, blue for carbon, and white for hydrogen.

The stable structural configuration of CO adsorbed on different sites in the case of a low CO coverage and in the context of the embedded cluster model are presented in **Figure** **10** (side view) and **11** (top view), as well as in Figure S1, Supporting Information. In these figures, it can be seen that CO binds to a surface Al^3+^ ion at *θ*[H_2_O] = 0 ML clearly preferring a perpendicular orientation (see Table S2, Supporting Information for the three tested DFT functionals). This orientation is facilitated through charge transfer of 0.16 e^−^ from CO to the surface^[^ ^38^ ^]^ (Figure 10a and 11a, Table S3, Supporting Information). However, in the cases of *θ*[Al(OH)_3_] = 1 and *θ*[H_2_O] = 1 ML, the large distances between the CO molecule and the OH groups on the surface show that CO is weakly bound and mainly stabilized by weak electrostatic interaction with the surface hydroxyl groups (see Figure 10b,c and 11b,c, as well as Table S4, Supporting Information).

The interaction of the CO molecule with the α‐Al_2_O_3_(0001) surface leads to adsorbate‐induced relaxations. Thus, upon adsorption of CO on the *θ*[H_2_O] = 0 ML surface, the Al^3+^ ion of the surface layer moved slightly downwards. In all other cases, for example, for *θ*[Al(OH)_3_] = 1 and *θ*[H_2_O] = 1 ML, the Al^3+^ at the adsorption site moved upwards due to the OH^…^CO interaction (Table S5, Supporting Information). The CO bond length calculated using hybrid functionals showed a small contraction on the *θ*[H_2_O] = 0 ML surface with respect to that in the gas phase. By contrast, this distance remained constant on the *θ*[Al(OH)_3_] = 1 and *θ*[H_2_O] = 1 ML surfaces (Table S5, Supporting Information). These observations are in accordance with the periodic DFT calculations using the method PBE(D3) (Figure 4).

As mentioned above, the *θ*[Al(OH)_3_] = 1 and *θ*[H_2_O] = 1 ML surfaces represent a full saturation of the α‐Al_2_O_3_(0001) surface with OH groups, which does not correspond to the partial hydroxylation observed in the experiment. Thus, to mimic the low coverage of hydroxyl groups, one single H_2_O molecule was added on the dry *θ*[H_2_O] = 0 ML surface in dissociated form, that is, one hydroxyl group was attached to the central Al^3+^ ion, and one H atom was added to a neighboring O^2−^ ion (denoted as *θ*[[H_2_O = 0]+OH + H]). The adsorption of CO on the OH group was further studied with the *θ*[[H_2_O = 0]+OH+H] cluster surface model (see Figure 10d and 11d, Table 1). This configuration was also observed in the periodic calculations in the cases of low coverage, that is, [*θ*[Al(OH)_3_] = 1/9 ML; *θ*[CO] = 1/9 ML] (Figure 6). The interaction of the CO molecule with the OH groups on the *θ*[Al(OH)_3_] = 1 and *θ*[H_2_O] = 1 ML surfaces as well as on the *θ*[[H_2_O = 0] + OH + H] cluster surface model was investigated by comparing the distance between the O atom (denoted as O_(CO)_–H_(surface)_) and the C atom (denoted as C_(CO)_–H_(surface)_) with the H atom of the nearby hydroxyl groups (see Figure 11, Table S6, Supporting Information). The shortest calculated bond distance between C_(CO)_–H_(surface)_ for the *θ*[Al(OH)_3_] = 1, *θ*[H_2_O] = 1, and the *θ*[[H_2_O = 0] + OH + H] cluster surface model is 2.7, 2.3, and 2.3 Å, respectively, which is shorter than the sum of the van der Waals radii. On the contrary, the O_(CO)_–H_(surface)_ bond distance for *θ*[Al(OH)_3_] = 1 and *θ*[H_2_O] = 1 ML surfaces is >3 Å in most of the cases (longer than the sum of van der Waals radii).^[^ ^39^ ^]^ These results suggest the presence of a weak OH^…^CO hydrogen bond.

**Figure 12:**
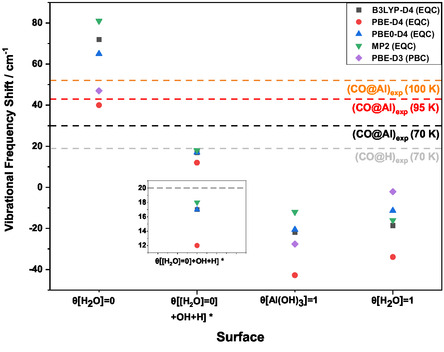
CO vibrational frequency shift relative to the gas‐phase CO value (2143 cm^−^^1^) obtained on various α‐Al_2_O_3_(0001) surfaces and calculated with different DFT functionals and the MP2 method in conjunction with the def2‐TZVPP basis set. Data using the EQC approach EQC and periodic DFT (PBC) with the PBE‐D3 functional are displayed. The horizontal lines mark the experimental data. The CO frequencies using the model *θ*[[H_2_O = 0] + OH + H] (see Section 3 for description) with CO adsorbed on the hydroxyl group (denoted *θ*[[H_2_O = 0] + OH + H]*) are also included. For *θ*[H_2_O] = 0 surface, the frequencies were calculated for the quantum cluster with shells relaxed until the 3rd shell, while for *θ*[Al(OH)_3_] = 1 ML, *θ*[H_2_O] = 1 ML, and *θ*[[H_2_O = 0] + OH + H] surfaces, frequencies were computed for the cluster with shells relaxed until 2nd shell.

**Figure** **12** shows the CO vibrational frequency shift with respect to the CO frequency in the gas phase calculated using the embedded quantum cluster (EQC) model together with periodic boundary conditions (PBC) computations, which are comparable with the “low‐coverage CO” character of the EQC calculations, that is, *θ*[CO] = 1/4 or 1/9 ML (the lowest coverages possible with the used unit cells). Data for the three analyzed surfaces (*θ*[H_2_O] = 0, *θ*[Al(OH)_3_] = 1, and *θ*[H_2_O] = 1 ML) with *θ*[CO] = 1/4, 1/9 ML as well as for the “low‐coverage OH” *θ*[[H_2_O = 0] + OH + H] model in conjunction with the experimental values at different temperatures are displayed. Since the theoretical data in Figure 12 are oriented to low‐coverage cases, they are especially comparable with the experimental data at 100 K in the case of CO adsorption on Al^3+^. The results reveal that the Δ[ν(CO)] calculated for the CO@Al^3+^ interaction on the *θ*[H_2_O] = 0 ML surface was significantly blue‐shifted, which is in good agreement with the experimental observation. The CO vibrational frequency shift is method‐dependent, with a larger shift observed for hybrid functionals and MP2 compared to the GGA functional. In contrast, a small red shift is observed for CO adsorption on the *θ*[Al(OH)_3_] = 1 and *θ*[H_2_O] = 1 ML surfaces.

## 3 Discussion

In this study, the key properties investigated by different computational methods and IR spectroscopy are the CO stretching frequency and the CO binding energies on α‐Al_2_O_3_(0001). In the experiments, XPS estimated a surface concentration of 18% OH groups. Therefore, we concentrate in the following on the calculations on the water‐free *θ*[H_2_O] = 0 ML surface and on the EQC *θ*[[H_2_O = 0] + OH + H] as well as the PBC calculations for (*θ*[Al(OH)_3_] = 1/9 ML). IRRAS data for CO adsorbed on the partially hydroxylated α‐Al_2_O_3_(0001) surface were measured at temperatures ranging from 70 to 100 K. The band assigned to CO bound to OH appears only at 70 K, while the Al^3+^‐related CO band shifts to higher frequencies as the temperature increases, corresponding to a decrease in CO coverage (see Figure 2). In the EQC calculations, only a single CO molecule is adsorbed, resembling the low CO coverage regime. Therefore, these values are always compared with the high‐temperature experimental value for the Al^3+^ site (2195 cm^−1^ at 100 K), where CO coverage is expected to be the lowest (Figure 2c). When comparing EQC and PBC data, the lowest possible CO coverages were always considered. In the PBC calculation, the CO coverage can be varied and compared with the temperature dependence of the experimental data. The PBC and EQC calculations reveal that the Δ[ν(CO)] for CO@Al^3+^ on the *θ*[H_2_O] = 0 surface is blue‐shifted, agreeing well with the experimental results across the temperature range, particularly at 100 K, where CO coverage is lowest (see Figure 12). A blue shift of the CO vibrational frequency adsorbed on bare oxide surfaces has also been observed in other cases such as in MgO(001) (14.3 cm^−1^, *θ* → 0 ML)^[^ ^40^, ^41^, ^42^ ^]^ and CeO_2_(111) (11 cm^−1^, 1 ML).^[^ ^43^, ^44^ ^]^ The frequency shift is always larger as the coverage decreases, consistent with previous experimental results and PBC calculations.^[^ ^29^, ^36^, ^40^, ^44^ ^]^ Special in the present case is the large magnitude of the blue shift, ranging from 29 cm^−1^ (70 K) to 52 cm^−1^ (100 K). All theoretical predictions in this work confirm this significantly large blue shift, although its magnitude varies depending on the approach and the DFT functional used (see Table 1 for more details). The periodic DFT calculations and EQC computations using the GGA functional PBE are close (by 7 cm^−^^1^) but smaller than the experimental value by 5 and 12 cm^−^^1^, respectively. This is in line with the results for CO on CeO_2_(111),^[^ ^43^, ^44^ ^]^ where the PBE+U data showed a much smaller blue shift (+1 cm^−1^) compared to the experimental result (+11 cm^−1^) or the HSE06 value (+14 cm^−1^). The latter is in much better agreement with the experiment. The hybrid functionals (B3LYP and PBE0) and the wavefunction‐based method MP2 showed larger frequency shifts. One reason could be that the latter values correspond to the adsorption of a single CO molecule, whereas the experimental coverage might still be higher. While the theoretical results are conclusive, it would be interesting for future work to compare them with periodic calculations using hybrid functionals (e.g., PBE0) or a functional based on screened Coulomb potential such as HSE06,^[^ ^45^, ^46^ ^]^ which has proven highly successful for CO adsorption on the low‐index cerium oxide surfaces.^[^ ^43^, ^44^ ^]^ Using those structures as starting points for the embedded cluster calculations could also affect the frequencies by a few cm^−1^ as observed in our previous work on CO adsorption on CeO_2_(111).^[^ ^47^ ^]^ The embedded cluster calculations can be further influenced by using larger basis sets and by replacing part of the ECP—68 of the 75 Al atoms of cluster 3 (Figure 8) by all electron atoms—which could alter the predicted CO vibrational frequency.

Regarding CO adsorption on hydroxyl groups, the CO signal is observed only at 70 K because the weak OH^…^CO. bond does not persist at higher temperatures (see Figure 2b, Table 1). In the experiment, a less pronounced blue shift of the CO vibrational frequency of 20 cm^−1^ is detected, consistent with those reported for CO adsorption on other hydroxylated oxide surfaces.^[^ ^31^, ^32^, ^33^, ^34^, ^48^ ^]^ In our calculations, a comparable blue shift is predicted for low hydroxyl coverage, both in PBC (*θ*[Al(OH)_3_] = 1/9 ML) and in EQC on the *θ*[[H_2_O = 0] + OH + H] surface. In contrast, frequency shifts close to zero or even red shifts are obtained in the calculations with high OH coverages, as seen on the *θ*[Al(OH)_3_] = 1 and *θ*[H_2_O] = 1 ML surfaces, for both PBC and EQC. These results support a low degree of hydroxylation of the α‐Al_2_O_3_(0001) surface observed in the experiment.

The adsorption/binding energy (BE) of CO on α‐Al_2_O_3_(0001) with varying degrees of hydroxylation was also investigated in this work. The experimental results were provided in Section 2.1, 3, and a comprehensive study using periodic DFT (PBC) was presented in Section 2.2.1 (Figure 3, 4, 5, 6, 7). The BSSE‐corrected binding energies obtained using the embedding quantum cluster (EQC) model are given in Table 1 for the water‐free *θ*[H_2_O] = 0 ML surface and the *θ*[[H_2_O = 0] + OH + H] model. An exact determination of the adsorption energy is a challenging task, both experimentally and theoretically.^[^ ^49^, ^50^ ^]^ In this work, for the two different types of CO species, CO–Al^3+^ and OH^…^CO, only a difference in the BE of 0.05 eV could be experimentally determined (see Section 2.1and Table 1). This difference is significantly smaller than that obtained in the PBC calculations (see Figure 4), where, regardless of CO coverage and surface hydroxylation, the BE of CO on Al^3+^ sites is about −0.6–−0.8 eV while it is less than −0.4 eV for CO on hydroxyl groups. In the context of the EQC model, the BSSE is a challenge. The CP scheme is recognized to be approximate and frequently converges only at the basis set limit.^[^ ^51^, ^52^, ^53^ ^]^ In the present work and in the case of CO (low coverage, *θ*[CO] = 1/4,1/9 ML) adsorbed on the bare α‐Al_2_O_3_(0001) surface (*θ*[H_2_O] = 0 ML), the CP correction represents 45%–55% of the BE, which is a very large contribution difficult to be addressed accurately. A similar case was recently reported for CO adsorption on CeO_2_(111).^[^ ^47^ ^]^ In the present work, the defined clusters contain many ECPs for Al and a small basis set (def2‐SVP) for part of the oxygen atoms located further from the adsorption site. Consequently, reaching the basis set limit^[^ ^53^ ^]^ proves to be a troublesome endeavor. Table 1 reports our current best estimate for the BE. These values are in good agreement with the PBC computations, particularly when using the hybrid functional PBE0 (see Table 1). However, a more detailed study is required to identify different sources of errors in details. Under the low‐coverage OH and CO conditions denoted as *θ*[[H_2_O = 0] + OH + H], that is, *θ*[Al(OH)_3_] = 1/9 ML and *θ*[CO] = 1/9 ML in the PBC context, the CP correction accounts for only 10%–17% of the BE, resulting in values around −0.23 eV (see Table 1). These results agree well with the experimental and PBC data, supporting the setup of the embedded cluster approach, which enabled the use of hybrid functionals and wavefunction‐based methods.

## 4 Conclusions

In this work, we presented a systematic investigation of the surface structure and reactivity of the α‐Al_2_O_3_(0001) single‐crystal surface using a multitechnique approach that combines experiment (polarization‐resolved IRRAS and grazing‐emission XPS) and theory (periodic DFT calculations and embedded cluster computations). The results consistently demonstrated that the pristine, Al‐terminated α‐Al_2_O_3_(0001) surface is highly active and partially hydroxylated even under UHV conditions. The occurrence of partial hydroxylation was identified by two characteristic CO bands at 2172–2195 and 2163 cm^−1^, which are assigned to CO molecules bound respectively to surface Al^3+^ and OH groups (the latter via OH^…^CO hydrogen bonding) with distinct binding energies. The periodic DFT and embedded cluster model calculations showed that the CO vibrational frequency is more significantly blue‐shifted for adsorption on Al^3+^ than on OH groups. The higher the CO coverage, the smaller the observed frequency shift. There is good agreement in the CO vibrational frequency, BE, and adsorption configuration between experimental data and computed results obtained using both periodic DFT and embedded quantum cluster models.

## 5 Experimental Section

5.1

5.1.1

##### Experimental Methods

The polarization‐resolved IRRAS measurements were conducted in a sophisticated UHV apparatus “THEO” combining a state‐of‐the‐art FTIR spectrometer (Bruker Vertex 80v) with a multichamber UHV system (Prevac). This apparatus was dedicated to IR spectroscopic investigations on metal oxide systems in the form of both well‐defined single crystals (polarization‐ and azimuth‐resolved IRRAS) and nanostructured powders (temperature‐dependent IR transmission, UHV‐FTIRS).^[^ ^23^, ^54^ ^]^ The α‐Al_2_O_3_(0001) single crystal (10 × 10 × 1 mm) was purchased from Crystal GmbH. Firstly, the α‐Al_2_O_3_(0001) surface was cleaned by repeated cycles of sputtering with 2 keV, 4 mA, and 3×10^−6^ mbar Ar^+^ for 10 min and annealing at 850 K for 5 min in an O_2_ atmosphere of 1 × 10^−7^ mbar, subsequently annealing at 850 K without O_2_ for 5 min. The cleanliness and oxidation states of the samples were monitored by grazing incidence XPS equipped with a VG Scienta R4000 electron energy analyzer.

Polarization‐resolved IRRAS spectra were recorded with both s‐ and p‐polarized IR light at a fixed grazing incidence of 80°. Exposure to CO at sample temperatures typically below 80 K (using liquid He) was achieved by backfilling the IR chamber with a leak‐valve‐based directional doser, which was connected to a tube (2 mm in diameter) positioned 3 cm from the sample surface and 50 cm from the hot‐cathode ionization gauge. The base pressure during IR data acquisition was below 1 × 10^−10^ mbar. Prior to each exposure, a spectrum of the clean sample surface was recorded as a background reference. All IRRAS data shown here were difference spectra obtained by subtracting the reference. All IR spectra were acquired by recording 1024 scans with a resolution of 4 cm^−1^. Exposures were given in units of Langmuir (L) (1 L = 1.33 × 10^−6^ mbar s).

##### Computational Details: Periodic DFT Calculations

All periodic DFT calculations were performed with the Vienna Ab‐initio Simulation Package (VASP) version 5.4.1^[^ ^55^, ^56^ ^]^ and the Atomic Simulation Environment^[^ ^57^ ^]^ using the PBE functional^[^ ^58^ ^]^ with Grimme's D3 dispersion correction^[^ ^59^ ^]^ and the projector‐augmented wave method.^[^ ^60^, ^61^ ^]^ The lattice constants of *α*‐Al_2_O_3_ were taken from a previous study on the hydroxylation of *α*‐Al_2_O_3_.^[^ ^37^ ^]^ Slabs calculations were performed with a cutoff of 400 eV and a Γ‐centered k‐point grid with a 4 × 4 × 1 mesh for the (1 × 1) surface slab unit.

Surfaces were modeled as slabs with seven formula units of Al_2_O_3_ per stoichiometric (1 × 1) surface and slabs of corresponding thickness for other terminations. The lower part of the slabs was terminated by a single Al layer (stoichiometric termination), and the bottom 3.5 formula units of Al_2_O_3_ per (1 × 1) surface were kept frozen at their bulk positions. The slabs were separated by at least 16 Å of vacuum to prevent artificial interaction between periodic images. In the CO adsorption calculations, a plane wave basis set was used for the electronic density, which included reciprocal lattice vectors with a norm up to 3*/*2 or 2 times larger than for the wave function, |*G*_cut_| (PREC = Normal or Accurate in VASP). CO and binding atoms (Al or OH) were included in the numerical Hessian, with a displacement of 0.01 or 0.02 Å. The calculations were performed with real‐space projectors (LREAL = AUTO), and the self‐consistent field (SCF) procedure was converged to a threshold of 10^−8^ eV for the total energy. A criterion of 0.005 eV Å^−1^ for the maximum forces on individual atoms was used for geometry convergence. Gaussian smearing with a 0.1 eV width was applied.

##### Embedding Cluster Computations

The embedded cluster calculations were carried out with the TURBOMOLE package^[^ ^62^ ^]^ along with the RI‐J (resolution of identity for the Coulomb term J)^[^ ^63^ ^]^ approximation on the structural information obtained in Ref.^[^ ^37^ ^]^ where surface hydroxylation for α‐Al_2_O_3_(0001) was investigated by periodic DFT calculations. Three different terminations from ref.^[^ ^37^ ^]^ were considered: the water‐free surface *θ*[H_2_O] = 0, fully hydroxylated surface *θ*[Al(OH)_3_] = 1, and one with dissociated H_2_O (*θ*[H_2_O] = 1). In all cases, only one CO molecule was adsorbed at the surface, resembling best the low CO coverage case in the experiment and the lowest coverages in the periodic DFT calculations *θ*[CO] = 1/9 ML or *θ*[CO] = 1/4 ML. In the first step, a point charge field (PCF) was generated with the nominal charges +3 for Al, −2 for O, and +1 for H. Setting up the PCF directly from the abovementioned structures led to a dipole moment perpendicular to the surface. This was a technical issue in the embedded cluster calculations; additional charges above the surface at some distance to the adsorption site had to be added to compensate for the dipole moments. For *θ*[H_2_O] = 0, this problem was handled by using the structure of the fully optimized slab from ref.^[^ ^37^ ^]^ Therefore, the fully relaxed structure was used in the latter case, while for the *θ*[Al(OH)_3_] = 1 and *θ*[H_2_O] = 1 surfaces, those structures were chosen where only the upper layers of the slabs were optimized. At the adsorption site and in its neighborhood, the point charges were replaced by a quantum cluster (QM). Clusters of different sizes and shapes were constructed.^[^ ^64^ ^]^ The outermost shell consisted always of Al^3+^ ions described by effective core potentials (ECP)^[^ ^65^ ^]^ without a basis set to avoid fluctuation of electrons into the PCF. The QM cluster was treated using DFT with three different functionals: PBE,^[^ ^58^ ^]^ B3LYP,^[^ ^66^ ^]^ and PBE0^[^ ^67^ ^]^ in conjunction with the def2‐SVP and def2‐TZVPP basis sets.^[^ ^68^ ^]^ The def2‐TZVPP basis set was used for the parts of the cluster that were relaxed in the geometry optimization, while for the parts kept fixed, the def2‐SVP was used for the oxygen atoms, and the ecp‐10 Hay & Wadt effective core potential was utilized for the 10 core electrons of the Al^3+^ ions.^[^ ^65^ ^]^ Dispersion corrections were included using the D4 model.^[^ ^69^ ^]^ When not stated otherwise, the default parameters of TURBOMOLE were used for the geometry optimization and computation of the vibrational frequencies. Additionally, second‐order Møller–Plesset perturbation theory (MP2)^[^ ^70^ ^]^ was also used for some calculations. The CO harmonic vibrational frequency obtained with each method was scaled using a scale factor (*λ*) defined as $\lambda =\frac{\upsilon_{\text{CO}}^{\text{exp}}}{\omega_{\text{CO}}^{\text{cal}}}$, where $\upsilon_{\text{CO}}^{\text{exp}}$ was the experimental frequency for the CO molecule in the gas phase (2143 cm^−1^),^[^ ^71^ ^]^ and $\omega_{\text{CO}}^{\text{cal}}$ was the harmonic vibrational frequency of the CO(g) molecule computed with the corresponding theoretical method.

The adsorption/binding energies of a molecule **M** on a surface **S** were calculated according to the following formula

(1) $$E_{\text{ads}}=E[M@S]-E[S]-E[M]$$

where *E*[**M**@**S**], *E*[**S**], and *E*[**M**] were the energies of the surface with the adsorbed CO, the surface alone, and the energy of the CO molecule in the gas phase in the structures optimized for the respective systems. Because of the incompleteness of the basis set, these energies had to be corrected for the basis set superposition error (BSSE) using the full counterpoise (CP) scheme.^[^ ^72^ ^]^ The final binding energy (BE) is given by

(2) $$E_{\text{ads+CP}}=E_{\text{ads}}+\Delta E[S]+\Delta E[M]$$

where $\Delta E[S]=E[S_{\text{M@S}}]-E_{\text{ghost}}[S_{\text{M@S}}]$ and $\Delta E[M]=E[M_{\text{M@S}}]-E_{\text{ghost}}[M_{\text{M@S}}]$ were the energy differences of the fragments without and with the basis set of the other fragment (indicated by ghost) at the structures they have in the full system **M**@**S**.

## Conflict of Interest

The authors declare no conflict of interest.

## Supporting information

Supplementary Material

## Data Availability

The data that support the findings of this study are available in KITOpen at http://doi.org/10.35097/sg9tcyz24sr4cv77. The data are also available from the corresponding authors upon reasonable request.
