# Supplementary material for: Adsorption of CO on α‐Al2O3(0001): A Combined Experimental and Computational Study
Source: Chemphyschem. 2025 Apr 17;26(10):e202401134. doi: 10.1002/cphc.202401134 (PMC12091847; doi:10.1002/cphc.202401134)
Supplement: Supplementary file 1 — Supplementary Material [file CPHC-26-e202401134-s001.pdf]

Supplementary Information

Adsorption of CO on α-Al_2O_3(0001): A combined experimental and computational study

Siddhi Gojare,[1,[+]] Shuang Chen,[2,[+]] Jiachen Chen,[3,[+]] Zairan Yu,[2] Juana Vázquez Quesada,[1] Philipp N. Pleßow,[3,*] Karin Fink,[1,*] and Yuemin Wang[2,*]

[1]Institute of Nanotechnology (INT), Karlsruher Institut für Technologie (KIT), Kaiserstraße 12, 76131 Karlsruhe, Germany.)

[2]Institute of Functional Interfaces (IFG), Karlsruhe Institute of Technology (KIT), Kaiserstraße 12, 76131 Karlsruhe, Germany.

[3]Institute of Catalysis Research and Technology (IKFT), Karlsruhe Institute of Technology (KIT), Kaiserstraße 12, 76131 Karlsruhe, Germany.

[+] These authors contributed equally to this work.

*Corresponding authors: philipp.plessow@kit.edu; karin.fink@kit.edu; yuemin.wang@kit.edu

Embedded cluster DFT calculations

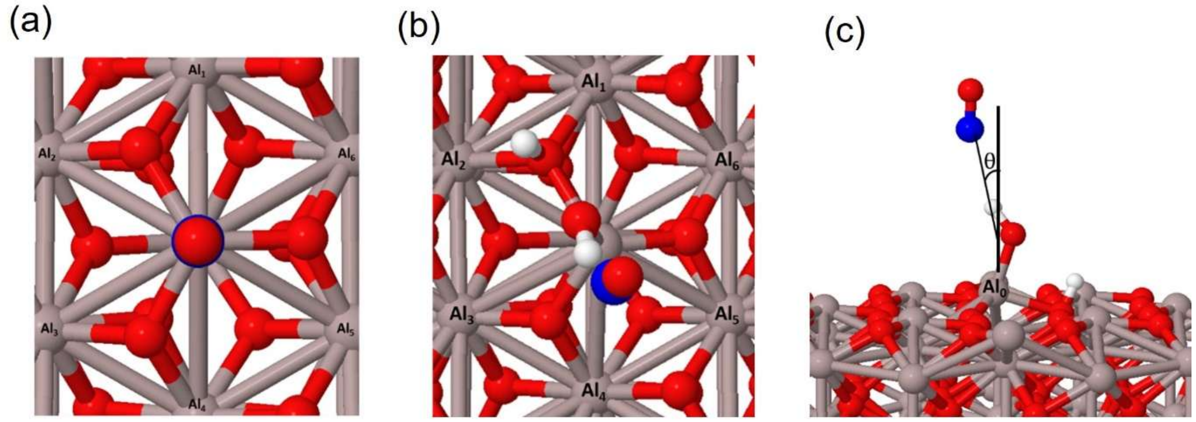

Figure S1. Schematic of CO adsorbed on (a) θ[H_2O]=0 ML and (b-c) θ[[H_2O=0]+OH+H] α-Al_2O_3(0001) surfaces showing the nearby atoms considered for calculating the CO tilt angle.

Table S1. CO binding energy (E_{bind}), scaled CO vibrational frequency (v_{CO}) on the clusters of θ[H_2O]=0 ML (water-free/dry/clean) α-Al_2O_3(0001) surface, calculated using the B3LYP/def2-TZVPP level of theory (no BSSE correction included).

Cluster size	E_{bind} (eV)	V_{co} (cm^{-1})
(1)	-0.56	2210
(2)	-0.75	2165
(3)	-0.95	2195
(4)	-0.97	2192

Table S2 Tilt angle of CO adsorption relative to the z-axis on α-Al_2O_3(0001) surfaces at θ[H_2O]=0 ML and θ[[H_2O=0]+OH+H], calculated with respect to the nearby atoms in the cluster (refer **Figure 10** in the manuscript and **Figure S1**).

Atoms selected	CO tilt angle (θ) (°)					
	θ[H_2O]=0			Θ[[H_2O=0]+OH+H]		
	PBE	B3LYP	PBE0	PBE	B3LYP	PBE0
Al_0-C-O	0	0	0	51	13	43
Al_1-C-O	40	40	40	66	35	57
Al_2-C-O	37	37	38	71	33	65
Al_3-C-O	40	40	40	60	25	57
Al_4-C-O	38	37	38	31	13	34
Al_5-C-O	40	40	40	24	17	16
Al_6-C-O	38	37	38	45	27	35
O_1-C-O	34	34	34	59	24	51
O_2-C-O	20	20	20	64	24	58
O_3-C-O	34	34	34	47	11	43
O_4-C-O	20	20	20	33	6	27
O_5-C-O	34	34	34	38	10	30
O_6-C-O	20	20	20	55	27	46

Table S3. Charges computed using Natural Orbital Population analysis on C, Al, O atoms of the θ[H_2O]=0 ML α-Al_2O_3(0001) surface using the B3LYP/def2-TZVPP level of theory.

Model	Atoms	Charge	Orbital population	
			Core	Valence
Surface	Al	2.22036	9.99151	0.73007
$(CO)_g$	C	0.4859	1.99977	3.46658
	O	-0.4859	1.99976	6.45031
CO on θ[H_2O]=0	Al	2.04789	9.99174	0.90611
	C	0.61158	1.99959	3.34254
	O	-0.38289	1.99976	6.35025

Table S4. Bond length of CO molecule computed at the def2-TZVPP level of theory.

Surface	Al-CO (Å)				CO (Å)			
	PBE	B3LYP	PBE0	MP2	PBE	B3LYP	PBE0	MP2
θ[H_2O]=0	2.14	2.17	2.16	2.18	1.13	1.12	1.12	1.13
θ[$Al(OH)_3$]=1	3.47	3.56	3.53	3.57	1.14	1.13	1.13	1.14
θ[H_2O]=1	4.24	4.32	4.27	4.36	1.14	1.13	1.13	1.14
$(CO)_g$	-	-	-	-	1.14	1.13	1.12	1.14

Table S5. The adsorbate-induced relaxation value (Å) (the change in the distance between the central Al and the bottom Al atom upon CO adsorption).

Relaxed shell	Surface	Layer relaxation (Å)		
		PBE	B3LYP	PBE0
1st	Water free	-0.29	-0.24	-0.27
	Fully hydroxylated	-0.04	-0.04	-0.05
	H_2O dissociated	-0.19	-0.17	-0.19
2nd	Water free	-0.09	-0.02	-0.06
	Fully hydroxylated	+0.04	+0.03	+0.03
	H_2O dissociated	+0.09	+0.07	+0.08
3rd	Water free	-0.11	-0.03	-0.08
	Fully hydroxylated	+0.01	0	0
	H_2O dissociated	+0.05	+0.04	+0.03
4th	Water free	+0.36	+0.16	+0.24
	Fully hydroxylated	0	+0.02	0
	H_2O dissociated	+0.04	+0.02	+0.03

Table S6. The bond length (Å) between CO molecule and surface OH group (denoted as O_{CO}-$H_{surface}$ and C_{CO}-$H_{surface}$) present on θ[[H_2O=0]+OH+H], θ{$Al(OH)_3$]=1 ML, θ{H_2O]=1 ML surfaces. Change in OH bond length before CO adsorption (denoted as ($OH_{surface}$) and after CO adsorption (denoted as $OH_{surface}$/CO). Everything calculated at the def2-TZVPP level of theory.

Surface	Atoms (X: O_{CO} / C_{CO})	O_{CO}-$H_{surface}$ (Å)			C_{CO}-$H_{surface}$ (Å)			$OH_{surface}$/CO ($OH_{surface}$) (Å)		
		PBE	B3LYP	PBE0	PBE	B3LYP	PBE0	PBE	B3LYP	PBE0
θ[$Al(OH)_3$]=1	X-H_1	2.62	2.61	2.64	3.18	3.23	3.25	0.97 (0.97)	0.97 (0.97)	0.97 (0.97)
	X-H_2	3.11	3.06	3.09	3.09	3.1	3.12	0.98 (0.98)	0.97 (0.97)	0.97 (0.97)
	X-H_3	3.78	3.77	3.74	2.68	2.72	2.68	0.97 (0.97)	0.96 (0.96)	0.96 (0.96)
	X-H_4	3.56	3.53	3.55	3.12	3.16	3.17	0.98 (0.97)	0.96 (0.96)	0.96 (0.96)
θ[H_2O]=1	X-H_1	3.17	3.17	3.14	3.15	3.17	3.19	0.97 (0.97)	0.97 (0.97)	0.97 (0.97)
	X-H_2	3.67	3.74	3.7	2.97	3.1	3.12	0.98 (0.98)	0.98 (0.98)	0.98 (0.98)
	X-H_3	3.17	3.23	3.23	3.13	3.21	3.24	0.97 (0.97)	0.97 (0.97)	0.97 (0.97)
	X-H_4	3.24	3.32	3.33	2.24	2.29	2.27	0.97 (0.97)	0.96 (0.96)	0.96 (0.96)
θ[[H_2O=0]+ OH+H]	X-H	3.25	3.42	3.31	2.32	2.33	2.33	0.97 (0.96)	0.96 (0.96)	0.95 (0.95)

Table S7. Periodic DFT results of CO adsorption energy (E_{ads}), shifted CO vibrational frequency (ν_{CO}), and distances calculated for C-O bond of CO molecule.

Surfaces				Calculated Value			VASP Setting		
θ[$Al(OH)_3$]	θ[H_2O]	θ[CO]	Site	E_{ads} / eV	ν_{CO} / cm^{-1}	$d_{C\text{-}O}$ / Å	PREC	NFREE	δ / Å
0	0	1/4	Al	-0.685	47	1.138	normal	2	0.01
0	0	1/2	Al	-0.656	29.7	1.141	accurate	4	0.02
0	0	1/2	Al	-0.655	31.7	1.141	accurate	2	0.01
0	0	3/4	Al	-0.635	17.8	1.142	normal	2	0.01
0	0	3/4	Al	-0.635	19.5	1.142	normal	2	0.01
0	0	1	Al	-0.613	7.3	1.144	normal	2	0.01
0	1/4	1/4	Al	-0.778	58.5	1.137	normal	2	0.01
0	1/4	1/4	H	-0.284	-0.5	1.144	normal	2	0.01
0	1/4	1/2	Al	-0.739	44.4	1.139	accurate	2	0.01
0	1/4	3/4	Al	-0.636	28.5	1.141	accurate	2	0.01
0	1/2	1/4	Al	-0.741	61.8	1.136	normal	2	0.01
0	1/2	1/4	Al	-0.73	60.1	1.137	normal	2	0.01
0	1/2	1/4	Al	-0.761	57.7	1.137	accurate	2	0.01
0	1/2	1/4	Al	-0.74	62.5	1.136	accurate	2	0.01

0	1/2	1/4	H	-0.243	-25.3	1.146	accurate	2	0.01
0	1/2	1/4	H	-0.262	-6	1.144	accurate	2	0.01
0	1/2	1/2	Al	-0.722	44.5	1.139	normal	2	0.01
0	1/2	1/2	Al	-0.656	43.3	1.139	accurate	2	0.01
0	1/2	1/2	Al	-0.653	41.7	1.139	accurate	2	0.01
0	1/2	1/2	H	-0.265	-11.3	1.145	accurate	2	0.01
0	1/2	1/2	H	-0.276	-28.6	1.147	accurate	2	0.01
0	3/4	1/4	Al	-0.716	60.2	1.137	accurate	2	0.01
0	3/4	1/4	H	-0.292	-0.1	1.144	accurate	2	0.01
0	1	1/4	H	-0.369	-2.1	1.144	normal	2	0.01
0	1	1/4	H	-0.329	-18.7	1.145	normal	2	0.01
0	1	1/4	H	-0.335	4.7	1.143	normal	2	0.01
0	1	1/2	H	-0.302	5.9	1.143	normal	2	0.01
0	1	1/2	H	-0.336	-1.1	1.144	normal	2	0.01
0	1	3/4	H	-0.304	5.5	1.143	normal	2	0.01
0	1	1	H	-0.306	6.2	1.143	normal	2	0.01
0	1	1	H	-0.215	-23.2	1.147	normal	2	0.01
1/9	0	1/9	Al	-0.646	30.7	1.140	normal	2	0.01
1/9	0	1/9	Al	-0.629	22	1.141	normal	2	0.01
1/9	0	1/9	Al	-0.639	40.3	1.139	normal	2	0.01

1/9	0	1/9	Al	-0.639	39.5	1.139	normal	2	0.01
1/9	0	1/9	H	-0.297	15.7	1.142	normal	2	0.01
1/9	0	2/9	H	-0.296	16	1.142	normal	2	0.01
1/9	0	1/3	H	-0.296	15.2	1.142	normal	2	0.01
1/4	0	1/4	H	-0.411	-0.4	1.144	normal	2	0.01
1/4	0	1/4	H	-0.419	15.5	1.142	normal	2	0.01
1/4	0	1/4	Al	-0.614	-9.1	1.144	normal	2	0.01
1/4	0	1/4	Al	-0.608	-5	1.143	normal	2	0.01
1/4	0	1/4	H	-0.411	2.9	1.143	normal	2	0.01
1/4	0	1/2	H	-0.316	10.5	1.142	accurate	2	0.01
1/4	0	1/2	H	-0.373	0.4	1.143	normal	2	0.01
1/4	0	3/4	H	-0.344	2.4	1.143	normal	2	0.01
1/4	0	3/4	H	-0.353	11.1	1.142	normal	2	0.01
1	0	1/4	H	-0.212	-15	1.145	normal	2	0.01
1	0	1/4	H	-0.13	5.6	1.143	normal	2	0.01
1	0	1/4	H	-0.235	-27.6	1.146	normal	2	0.01
1	0	1/4	H	-0.208	-24	1.146	normal	2	0.01
1	0	1/2	H	-0.25	-23.8	1.146	normal	2	0.01
1	0	1/2	H	-0.253	-24.4	1.146	normal	2	0.01
1	0	3/4	H	-0.261	-20.2	1.146	normal	2	0.01

1	0	1	H	-0.252	-11.7	1.145	normal	2	0.01
1	0	1	H	-0.269	-18.8	1.145	normal	2	0.01
